# Prolactin-induced protein mediates cell invasion and regulates integrin signaling in estrogen receptor-negative breast cancer

**DOI:** 10.1186/bcr3232

**Published:** 2012-07-20

**Authors:** Ali Naderi, Michelle Meyer

**Affiliations:** 1 The University of Queensland Diamantina Institute, Princess Alexandra Hospital, Ipswich Road, Brisbane Queensland 4102, Australia

## Abstract

**Introduction:**

Molecular apocrine is a subtype of estrogen receptor (ER)-negative breast cancer that is characterized by a steroid-response gene signature. We have recently identified a positive feedback loop between androgen receptor (AR) and extracellular signal-regulated kinase (ERK) signaling in this subtype. In this study, we investigated the transcriptional regulation of molecular apocrine genes by the AR-ERK feedback loop.

**Methods:**

The transcriptional effects of AR and ERK inhibition on molecular apocrine genes were assessed in cell lines. The most regulated gene in this process, prolactin-induced protein (*PIP*), was further studied using immunohistochemistry of breast tumors and xenograft models. The transcriptional regulation of PIP was assessed by luciferase reporter assay and chromatin immunoprecipitation. The functional significance of PIP in cell invasion and viability was assessed using siRNA knockdown experiments and the mechanism of PIP effect on integrin-β1 signaling was studied using immunoblotting and immunoprecipitation.

**Results:**

We found that *PIP *is the most regulated molecular apocrine gene by the AR-ERK feedback loop and is overexpressed in ER-/AR+ breast tumors. In addition, *PIP *expression is regulated by AR-ERK signaling in xenograft models. These observations are explained by the fact that *PIP *is a target gene of the ERK-CREB1 pathway and is also induced by AR activation. Furthermore, we demonstrated that PIP has a significant functional role in maintaining cell invasion and viability of molecular apocrine cells because of a positive regulatory effect on the Integrin-ERK and Integrin-Akt signaling pathways. In fact, PIP-knockdown markedly decreases the phosphorylation of ERK, Akt, and CREB1. Importantly, PIP knockdown leads to a marked reduction of integrin-β1 binding to ILK1 and ErbB2 that can be reversed by the addition of fibronectin fragments.

**Conclusions:**

We have identified a novel feedback loop between PIP and CREB1 mediated through the Integrin signaling pathway. In this process, PIP cleaves fibronectin to release fragments that activate integrin signaling, which in turn increases PIP expression through the ERK-CREB1 pathway. In addition, we demonstrated that PIP expression has a profound effect on cell invasion and the viability of molecular apocrine cells. Therefore, PIP signaling may be a potential therapeutic target in molecular apocrine breast cancer.

## Introduction

Estrogen receptor-negative (ER-) breast cancer is a heterogeneous disease that is characterized by an earlier time-to-relapse compared to ER+ breast tumors [1,2]. As opposed to ER+ breast cancer, where the estrogen receptor signaling has a critical biological and therapeutic role, there is limited knowledge available regarding the pathophysiology of ER- disease. Therefore, in order to discover effective therapeutic strategies in ER- breast cancer there is a need for better understanding of the biology of this disease.

ER- breast cancer can be divided into different molecular subgroups based on the expression microarray profiling [2-4]. The two most prominent ER- subgroups include molecular apocrine and basal subtypes [2-4]. The molecular apocrine subtype is characterized by a steroid-response gene signature that includes androgen receptor (AR), FOXA1, TFF3, and a high frequency of ErbB2 overexpression [3-5]. It is notable that AR expression is present in 40% to 50% of ER- breast tumors and the majority of these cases also have ErbB2 overexpression [2,6-8]. Furthermore, it has been suggested that a loss of PTEN at early stages of tumorigenesis predisposes to the formation of breast tumors with molecular apocrine features [9].

Over the past few years, several functional and genomic studies have signified the importance of AR and ErbB2 signaling in the biology of molecular apocrine breast cancer [2,5,10-13]. Notably, a recent meta-analysis study has revealed that AR and ErbB2 signaling are two major activated pathways in the molecular apocrine subtype [2]. In addition, we have previously demonstrated a functional cross-talk between the AR and ErbB2 signaling in molecular apocrine cells that modulates cell proliferation and expression of steroid-response genes [10]. Furthermore, other studies have shown that AR mediates ligand-dependent activation of the Wnt and ErbB2 signaling pathways through direct transcriptional induction of WNT7B and ErbB3 [12]. Importantly, AR signaling is a potential therapeutic target in ER-/AR+ breast cancer and is currently under investigation in a clinical trial (ClinicalTrials.gov Identifier: NCT00468715), [12,14-16].

To delineate the key signaling pathways involved in the biology of molecular apocrine breast cancer, we have recently identified a positive feedback loop between the AR and extracellular signal-regulated kinase (ERK) signaling pathways in this disease [11]. We have shown that in this feedback loop AR regulates ERK phosphorylation through the mediation of ErbB2 and, in turn, ERK-CREB1 signaling regulates the transcription of AR in molecular apocrine cells [11]. This feedback loop provides a molecular basis for the association between AR expression and the high prevalence of ErbB2 overexpression in molecular apocrine tumors [11]. In addition, it explains the mechanism for a synergistic response to the combination of AR and MEK inhibitors in molecular apocrine models [15]. Although published data support a significant biological role for the AR and ErbB2 signaling in molecular apocrine breast cancer, there is currently limited information regarding other functionally important genes and pathways in this disease.

In this study, we investigated the transcriptional regulation of top ranking genes in the molecular apocrine signature by the AR-ERK feedback loop. We discovered that Prolactin-Induced Protein (PIP) is highly regulated by this feedback loop. Importantly, we demonstrated that PIP is a key mediator of cell invasion and regulates integrin signaling in molecular apocrine cells.

## Materials and methods

### Cell culture and treatments

Breast cancer cell lines MDA-MB-453, HCC-1954, and MCF-7 were obtained from American Type Culture Collection (Manassas, VA, USA). All the culture media were obtained from Invitrogen (Melbourne, VIC, Australia). MDA-MB-453 and HCC-1954 cell lines were cultured in L15 medium, 10% fetal bovine serum (FBS) and RPMI 1640 medium, 10% FBS, respectively. The MCF-7 cell line was cultured in (D)MEM/F12 medium, 10% FBS. Cell cultures were carried out in a humidified 37°C incubator supplied with 5% CO_2_.

The following treatments were applied for the cell culture experiments: 1) AR inhibitor, flutamide (Sigma-Aldrich, Sydney, NSW, Australia) at 25 µM to 40 µM concentrations [11]; 2) MEK inhibitor, CI-1040 (PD184352), (Selleck Chemicals, Houston, TX, USA) at 2 µM to 10 µM concentrations [11]; and 3) 5α-androstan-17β-ol-3-one (dihydrotestosterone (DHT)), (Sigma-Aldrich) at 100 nM concentration [11,17]. Treatments with the inhibitors were performed in media containing FBS. DHT treatment was carried out in phenol red-free media (Invitrogen) with 10% Charcoal/Dextran treated serum (Thermo Scientific, Melbourne, VIC, Australia) and cell lines were cultured in the media for 48 hours prior to DHT treatment.

### Quantitative real-time polymerase chain reaction

Total RNA extraction was performed as described before [18]. Quantitative real-time PCR (qPCR) to assess the expression levels of PIP (assay ID: Hs00160082_m1), dual specificity phosphatase 6 (DUSP6, assay ID: Hs00737962_m1), S100A8 (assay ID: Hs00374264_g1), FOXA1 (assay ID: Hs00270129_m1), transcription factor AP2B (TFAP2, assay ID: Hs00231468_m1), SOX11 (assay ID: Hs00846583_s1), BANP (assay ID: Hs00215370_m1), PER2 (assay ID: Hs00256143_m1), TFF3 (assay ID: Hs00902278_m1), and AZGP1 (assay ID: Hs00426651_m1) was carried out using Taqman Gene Expression Assays (Applied Biosystems, Melbourne, VIC, Australia) as instructed by the manufacturer. Housekeeping gene RPLP0 (Applied Biosystems) was used as a control. Relative gene expression = gene expression in the knock-down group or following AR and MEK inhibition/average gene expression in the control group. Relative gene expression was calculated using the 2^-ΔΔCT ^formula as described before [19,20]. All experiments were performed in at least three biological replicates.

### Western blot analysis

PIP (GCDFP-15) rabbit monoclonal antibody (ab62363) was obtained from Abcam (Cambridge, UK). Rabbit monoclonal ERK1/2, rabbit monoclonal phospho-ERK1/2 (Thr202/Tyr204), rabbit monoclonal Akt (pan), rabbit monoclonal phospho-Akt (Ser473), rabbit monoclonal CREB, rabbit monoclonal phospho-CREB (Ser133), rabbit monoclonal ILK1, and rabbit polyclonal ErbB2 antibodies were obtained from Cell Signaling (Danvers, MA, USA). Rabbit polyclonal integrin-β1 antibody was obtained from Merck Millipore (Melbourne, VIC, Australia).

Western blots were carried out at 1:1000 dilution of each primary antibody using 10 µg and 20 µg of cell lysates for the total and phospho-proteins, respectively. Protein concentrations from the cell isolates were measured using the BCA Protein Assay Kit (Thermo Scientific). Rabbit polyclonal α-tubulin antibody (Abcam) was used as the loading control. Analysis of band densities was performed using Bio-Profil Densitometer Software (Vilber Lourmat, Eberhardzel, Germany). All fold changes in band densities were measured relative to the control groups. Western blots were performed in two biological replicates and the average fold change is shown for each set of experiments.

### Immunohistochemistry

Immunohistochemistry (IHC) staining was performed using EnVision+ System-HRP (AEC), (DakoCytomation, Melbourne, VIC, Australia) following the manufacturer's instructions. Antigen retrieval was carried out using Target Retrieval Solution (DakoCytomation). AR rabbit polyclonal and PIP rabbit monoclonal antibodies were obtained from AbCam. Primary antibody incubations were carried out at 1:100 dilutions. Slides were counterstained with hematoxylin (Sigma-Aldrich) and mounted using Glycergel Mounting Medium (DakoCytomation). For IHC scoring, slides were examined using a light microscope (Nikon Instruments Inc., Tokyo, Japan). A total of 1,000 cells per each slide were counted at 60X magnification to assess the percentage of cells showing positive staining for each antibody.

### Primary breast tumors

The Princess Alexandra Hospital human research ethics committee approved this study and informed consent was obtained from each patient for the use of tissue samples. A total of twenty-four paraffin-embedded ER- breast tumor samples were obtained from the Princess Alexandra Hospital tissue bank. IHC staining for AR and PIP were carried out as described above. For downstream analysis, tumors were classified into two groups based on their AR staining pattern as published before [11]: 1) AR+ group with ≥20% of cells showing positive AR staining, and 2) AR- group with <20% of cells stained for AR.

### Tumor xenograft studies

The University of Queensland animal ethics approval was obtained for the project and mice were maintained in accordance with the University of Queensland animal care guidelines. Xenograft studies were carried out as we previously published [11]. In summary, a total of 5 × 10^6 ^MDA-MB-453 cells were injected into the flank of each six-week-old female non-obese diabetic/severe combined immunodeficient mouse to generate the xenograft tumors. Treatments were initiated seven days after the cell injections.

Flutamide treatment was carried out with 25 mg/60-day slow-release flutamide pellets (Innovative Research of America, Sarasota, FL, USA) and MEK inhibition was carried out with daily oral gavage of MEK inhibitor PD0325901 (Selleck Chemicals) at 15 mg/kg/day as described before [11,21]. A total of four mice were studied in each of the following groups: 1) Control group received placebo pellets (Innovative Research of America) and daily oral gavage of an equal volume of carrier solution to that of the MEK inhibitor treatment group; 2) flutamide group was treated with the flutamide pellets and daily oral gavage of carrier solution; and 3) MEK inhibitor group had placebo pellets and daily oral gavage of PD0325901. Xenograft tumors were harvested 28 days following the start of treatment in each group. The harvested tumors were fixed in formalin and embedded in paraffin for IHC staining.

### Luciferase reporter assays

Full-length cDNA clones for CREB1 and AR were obtained from Open Biosystems (Thermo Scientific). The human prolactin receptor (PRLR) clone was obtained from GeneCopoeia (Rockville, MD, USA). The clones were validated by restriction digestion/sequencing and then sub-cloned in a pcDNA3.1 vector (Invitrogen) to generate expression constructs. Furthermore, the sequence of 1.5 kb promoter region of the *PIP *gene was obtained using Ensembl Genome Browser [22], and PCR-generated using the following primers: Forward-primer, 5'TCTTCTGCCTTATGCCTGCCTTGGT and Reverse-primer, 5'AAGTGGTGTCCCAGGTGCCCAG. PIP promoter was then cloned in a pGL3 luciferase reporter vector (Promega, Sydney, NSW, Australia) and validated by restriction digestion/sequencing.

To carry out the reporter assays, MCF-7 cells were co-transfected with the PIP reporter vector and expression vectors using ExGen 500 reagent (Fermentas Life Sciences, St Leon-Rot, Germany). The *Renilla *pRL-TK vector (Promega) was used as an internal control reporter. Co-transfection with PIP reporter vector and an empty pcDNA vector was used as a control. Forty-eight hours after the transfections reporter activities were measured using the Dual-Glo Luciferase Assay System (Promega) in an Orion II Microplate Luminometer (Berthold Detection Systems, Pforzheim, Germany). The response ratios for transcription factors and control were measured relative to the internal control reporter (relative response ratio). Reporter assay experiments were carried out in phenol red-free (D)MEM/F12 medium with 10% Charcoal/Dextran treated serum supplemented with 100 nM of DHT and 5 µg/ml of ovine prolactin (PRL, Sigma-Aldrich) [11,23]. All reporter assays were performed in four biological replicates

### **Chromatin immunoprecipitation assay**

Chromatin immunoprecipitation (ChIP) assays were performed in the MDA-MB-453 cell line using a ChIP Assay Kit (USB Corporation, Cleveland, OH, USA) as instructed by the manufacturer [24]. ChIP-grade rabbit monoclonal CREB1 (ChIPAb+ CREB kit, Millipore, Melbourne, VIC, Australia) antibody was applied at 4 µg per assay. To quantify ChIP results, two primer sets for PIP promoter were used for qPCR amplification using the SYBR green method (Applied Biosystems). Forward primer set 1: 5'AGTGGGGAGGGTGAATGGGTGAT (start: -141) and Reverse primer set 1: 5'AGTGCAAGAGCTGCCAGGGAGAT (start -85), Forward primer set 2: 5'TAAGCCAGCTCTTTGGTGCCAAG (start: -810) and Reverse primer set 2: 5'AGATCCCCCAGCTGCCCCACAAT (start -697). Amplification of input chromatin prior to immunoprecipitation (IP) at a dilution of 1:100 was used as a positive control. ChIP assays using non-specific antibody (rabbit IgG) served as a negative control. The assays were carried out in three replicates and percentage recovery of input chromatin was calculated for each experimental set.

### PIP siRNA knockdown

PIP-knockdown was carried out in MDA-MB-453 cells by reverse transfection as described before [25], using the following two sets of siRNA oligos (duplex, Sigma-Aldrich): Set 1: D1, 5'CCUAUGUGACGACAAUCCA; D2, 5'UGGAUUGUCGUCACAUAGG and set 2: D1, 5'CUCUACAAGGUGCAUUUAA; D2, 5'UUAAAUGCACCUUGUAGAG. CREB1-knockdown was carried out using the following siRNA oligo as described before: 5'GGUGGAAAAUGGACUGGCUtt [26]. Transfection of siRNA oligos using Lipofectamine RNAiMAX (Invitrogen) was carried out as instructed by the manufacturer. The final siRNA concentration was 20 nM for the knock-down experiments. Cells transfected with siRNA Universal Negative Control # 1 (non-targeting siRNA, Sigma-Aldrich) were used as controls. In all experiments the effects of knockdowns were assessed seventy-two hours after the siRNA transfections.

### Cell invasion assay

Cell invasion assays were carried out using CytoSelect 96-Well Cell Invasion Assay Kit (Cell Biolabs Inc., San Diego, CA, USA) as instructed by the manufacturer. PIP-siRNA and control-siRNA transfections were carried out in the MDA-MB-453 cell line as described before. Forty-eight hours after the siRNA transfections, cells were harvested and seeded in an invasion assay plate at 50,000 cells/100 µl per each well. Serum-free L15 medium and L15 medium with 10% FBS were used for the upper and lower chambers of the invasion assay plate, respectively. Next, cells were incubated for 24 hours in a 37ºC incubator before assaying for invasion. Finally, cells were dissociated from the membrane as per the manufacturer's instructions and subsequently detected with CyQuant GR Fluorescent Dye (Invitrogen). Fluorescence was measured with a fluorescence plate reader at 480 mm/520 mm. Treatment with Purified Human Fibronectin (α-Chymotryptic Fragment 120K, Merck Millipore) at 100 µg/ml concentration was carried out 24 hours after PIP-knockdown. Invasion assays were carried out in three biological replicates.

### Cell viability assay

PIP-knockdown in MDA-MB-453 cells was carried out as described before. A total of 10,000 cells transfected with either PIP-siRNA or control-siRNA were seeded per well of a 96-well plate. Seventy-two hours after transfections, cell viability was assessed using the Vybrant 3-(4,5-dimethythiazol-2-yl)-2,5-diphenyl tetrazolium bromide (MTT) Proliferation Assay Kit (Invitrogen) as instructed by the manufacturer. MTT assays were performed in eight biological replicates and absorbance at 570 nm was measured using a plate reader.

### Immunoprecipitation

Immunoprecipitation (IP) of integrin-β1 was carried out as previously published [27]. PIP-knockdown in the MDA-MB-453 cell line was performed in 6 cm dishes. Seventy-two hours after siRNA transfections, cells were lysed by 500 µl/per dish of 15 mM CHAPS (Sigma-Aldrich) in lysis buffer (0.15 M NaCl, 2mM EDTA, 1 mM PMSF, 1 mM NaVO4, 50 mM Tris-HCl, pH 7.5), then lysates were centrifuged for 20 minutes at 15,000 *g*. Next, the supernatants were pre-cleared with Protein A-Sepharose 4B beads (Invitrogen) for one hour and protein concentrations from the cell isolates were measured using the BCA Protein Assay Kit (Thermo Scientific).

Subsequently, we incubated 300 µg of each protein lysate with 4 µl of rabbit polyclonal integrin-β1 antibody (Merck Millipore, Cat. # AB1952) at 4ºC overnight followed by incubation with Protein A-Sepharose 4B beads at 4ºC for four hours. The Sepharose beads were washed three times with 15 mM CHAPS, then boiled for five minutes in SDS-PAGE sample buffer. Finally, samples were subjected to western blotting as described previously. Treatment with Purified Human Fibronectin (α-Chymotryptic Fragment 120K) at 100 µg/ml concentration was carried out 24 hours after PIP-knockdown. IP assays were performed in two biological replicates and the average fold change was shown for each set of experiments.

### Bioinformatics and statistical analysis

1) **Molecular apocrine-genes: **Top ranking genes in molecular apocrine-signature, based on their fold-change for gene expression, were extracted from a meta-analysis microarray study of 186 ER- breast tumors by Teschendorff *et al*. and an expression microarray study of ER- cell lines by Doane *et al*. [4,5]. The combination of the top eight genes in Teschendorff *et al.'*s study and the top six genes in Doane *et al*.'s study resulted in twelve unique molecular apocrine genes (Table 1).

**Table 1:** Fold changes of top ranking molecular apocrine-signature genes in two studies.

Gene	Fold change (Teschendorff *et al*., 2006)	Fold change (Doane *et al*., 2006)
FOXA1	2.55	7.8
TFAP2	2	44.7
BANP	1.9	N/A
S100A8	1.82	N/A
PER2	1.8	N/A
ErbB2	1.72	N/A
SOX11	1.71	N/A
DUSP6	1.66	N/A
AR	1.66	4.95
AZGP1	1.5	13.8
PIP	1.4	17.8
TFF3	1.11	6

N/A: not applicable

2) **Promoter analysis: **The sequences of the 1.5 kb promoter region of the *PIP *gene were obtained using Ensembl Genome Browser [22]. Identification of putative CREB1 binding sites in the promoter region was carried out using PATCH public 1.0 software [28].

3) **Bioinformantics and statistical analysis: **Heat map was generated using Spotfire DecisionSite for Functional Genomics (TIBCO, Somerville, MA, USA). Biostatistical analysis was carried out using IBM SPSS Statistics 20 (Armonk, NY, USA). The Mann-Whitney U test was applied for the comparison of non-parametric data. All error bars depict ± 2SEM.

## Results

### Molecular apocrine genes are regulated by AR-ERK signalling

To study the transcriptional regulation of key molecular apocrine genes by the AR-ERK feedback loop, we first identified the top ranking genes in the molecular apocrine signature based on their fold-change for gene expression as described in methods (Table 1). Among the top twelve genes in this ranking system, we have previously studied the transcriptional regulation of AR and ErbB2 in molecular apocrine breast cancer [10,11]. Therefore, in this study we investigated the remaining ten genes that are highly ranked in the molecular apocrine signature (Table 1).

Modulation of the AR-ERK feedback loop in molecular apocrine cell lines MDA-MB-453 and HCC-1954 was carried out using AR inhibitor flutamide and MEK inhibitor CI-1040 as we previously published [11,15]. Flutamide treatment was performed at 25 nM and 40 nM concentrations in MDA-MB-453 and HCC-1954 cell lines, respectively. These concentrations do not significantly inhibit ERK phosphorylation on their own; however, they have synergy with a low concentration of CI-1040 at 2 µM to inhibit ERK phosphorylation [11]. Furthermore, CI-1040 was applied at 2 µM and 10 µM, concentrations that result in a partial or complete inhibition of ERK phosphorylation, respectively [11]. Both cell lines were grown to 60% confluence and treated in the following groups: 1) control with vehicle only treatment, 2) CI-1040 at 2 µM, 3) flutamide treatments at 25 nM or 40 nM, 4) combination of CI-1040 at 2 µM and flutamide treatments, and 5) CI-1040 at 10 µM concentration. Forty-eight hours after the treatments, cells were harvested for RNA extraction and qPCR as described in methods.

**Figure 1:**
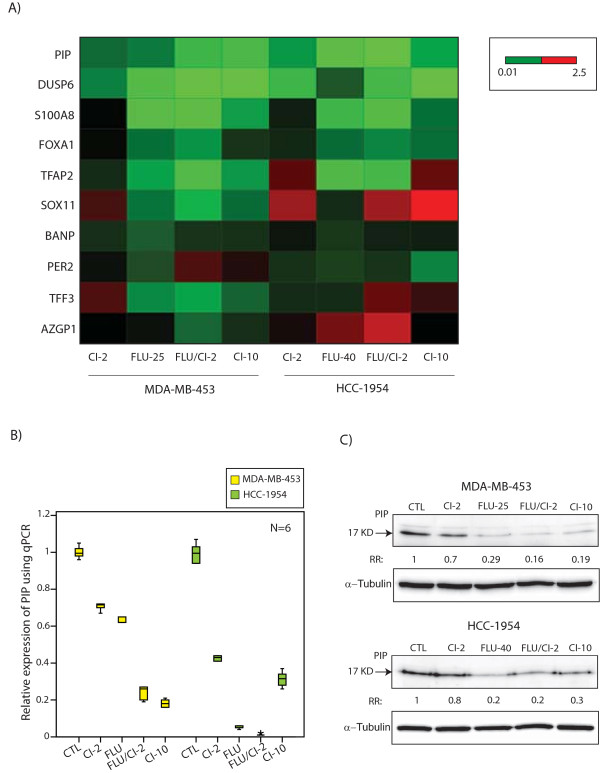
**The regulation of molecular apocrine genes by the AR-ERK feedback loop**. **(A) **Heat map of top ranking molecular apocrine-signature genes following the inhibition of AR-ERK signaling using qPCR data. Heat map shows fold changes for gene expression relative to control in MDA-MB-453 and HCC-1954 cell lines. Treatments were carried out by CI-1040 (CI) at 2 µM and 10 µM concentrations, flutamide (FLU) at 25 nM and 40 nM concentrations, and the combination of flutamide at 25 nM or 40 nM and CI-1040 at 2 µM concentrations. Red and green colors depict up-regulation and down-regulation, respectively. Bar indicates the range of fold changes in gene expression. **(B) **Box plots to demonstrate relative expression of PIP to control following AR-ERK inhibition in MDA-MB-453 and HCC-1954 cell lines using qPCR. CTL: control. **(C) **Western blot analysis to assess PIP protein levels following AR-ERK inhibition in MDA-MB-453 and HCC-1954 cell lines. Fold changes (RR) in band densities were measured relative to the control (CTL). AR, androgen receptor; ERK, extracellular signal-regulated kinase; qPCR, quantitative PCR; RR, relative risk.

The fold-changes for gene expression following treatments were calculated relative to that of the control group in both cell lines. Next, we ranked molecular apocrine genes based on their fold-change in expression following the modulation of AR-ERK signaling (Figure 1A and Table 2). We observed that *PIP*, *DUSP6*, *S100A8*, and *FOXA1 *expression were consistently reduced by the inhibition of AR and ERK as well as the combined inhibition of these two signaling pathways in both cell lines (Figure 1A and Table 2). The other molecular apocrine genes either did not have a consistent reduction or showed a slight increase in gene expression following the inhibition of AR and ERK (Figure 1A and Table 2). It is notable that CI-1040 at 2 µM concentration had markedly less effect compared to CI-1040 at 10 µM concentration (Table 2). Importantly, *PIP *and *DUSP6 *had the most prominent reduction in gene expression following the inhibition of AR-ERK with a fold-change ranging from 0.19 to 0.71 and 0.01 to 0.98, respectively (Figure 1A and Table 2). However, in contrast to *PIP*, flutamide treatment did not reduce *DUSP6 *expression in HCC-1954 cells (fold-change: 0.98). These data indicate that AR-ERK signaling regulates the transcription of selective molecular apocrine genes.

**Table 2:** Fold changes of molecular apocrine-signature genes following treatment with AR and MEK inhibitors.

Tx Gene	MDA-CI-2	MDA-FLU	MDA-FLU-CI	MDA-CI-10	HCC-CI-2	HCC-FLU	HCC-FLU-CI	HCC-CI-10
PIP	0.71	0.63	0.24	0.19	0.65	0.4	0.26	0.42
DUSP6	0.6	0.125	0.01	0.01	0.16	0.98	0.18	0.02
S100A8	1.2	0.09	0.06	0.45	1.3	0.86	0.77	0.68
FOXA1	1.2	0.67	0.5	0.9	1.08	0.71	0.58	0.68
TFAP2	1	0.43	0.15	0.5	1.61	0.53	0.68	1.69
SOX11	1.3	0.56	0.32	0.63	1.97	1	1.95	2.33
BANP	1	0.78	0.9	1	1.16	0.94	1.09	1.07
PER2	1.27	0.85	1.62	1.74	0.93	0.79	0.9	0.59
TFF3	1.71	0.55	0.43	0.73	1.23	1.23	1.72	1.43
AZGP1	1.36	1.29	0.73	1	1.3	1.78	2.23	1.21

* The average fold change to control for three biological replicate experiments is shown for each treatment group. Tx, treatment group; MDA, MDA-MB-453 and HCC, HCC-1954. FLU, flutamide at 25 µM and 40 µM concentrations for MDA-MB-453 and HCC-1954, respectively. CI-2, CI-1040 at 2 µM and CI-10: CI-1040 at 10 µM. Standard deviation of the fold changes: <0.05.

#### *PIP *expression is highly regulated by AR-ERK signaling

We observed that *PIP *expression was consistently reduced following the inhibition of AR-ERK signaling with a fold-change of 0.19 to 0.71 in MDA-MB-453 cell line and 0.26 to 0.65 in HCC-1954 line compared to the control groups (Table 2 and Figure 1B). We next examined the effect of AR-ERK inhibition on PIP protein level in MDA-MB-453 and HCC-1954 cell lines. Cells were harvested forty-eight hours after the treatments and PIP protein level was measured using western blot analysis. Notably, PIP protein levels were markedly reduced following AR-ERK inhibition with a fold-change of 0.16 to 0.7 and 0.2 to 0.8 compared to the control groups in MDA-MB-453 and HCC-1954 cell lines, respectively (Figure 1C). All together, our data suggest that *PIP *is significantly regulated by AR and ERK. Therefore, we investigated the biological significance of this gene in molecular apocrine breast cancer.

### PIP is overexpressed in ER-/AR+ primary breast tumors

**Figure 2:**
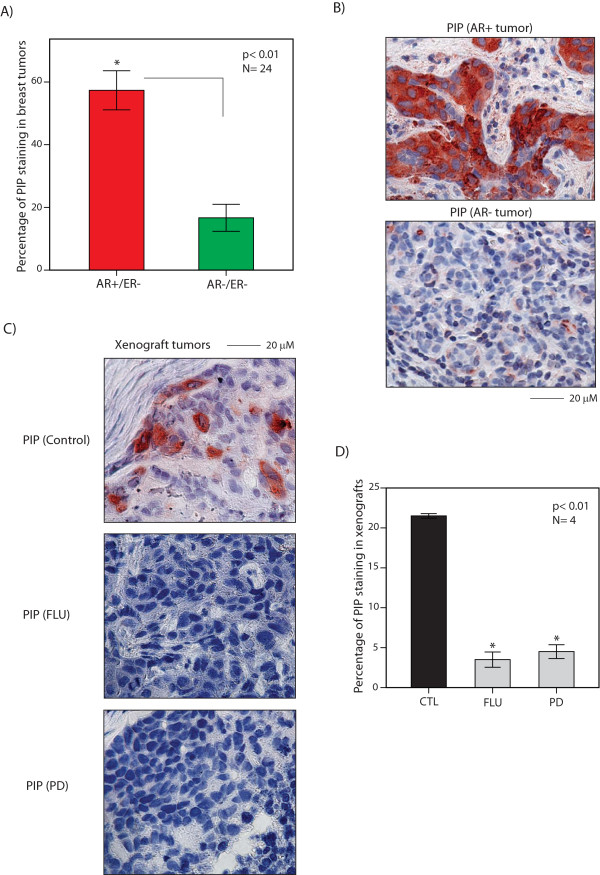
**PIP protein expression in primary breast tumors and *in vivo *models**. **(A) **Immunohistochemistry (IHC) staining for PIP in ER negative (ER-) breast tumors. AR+ group: ≥20% of cells showing positive AR staining; AR- group: <20% of cells stained for AR. Percentage of cells with positive staining are demonstrated for each group. *P <0.01 is for AR+ versus AR-. Error Bars: ± 2SEM. **(B) **IHC staining for PIP in AR+ and AR- breast tumors. Magnification is at 60X. **(C) **IHC staining for PIP in xenograft tumors generated using MDA-MB-453 cell line. Control: a control tumor; FLU: a flutamide-treated tumor; PD: a PD0325901-treated tumor. Magnification is at 60X. **(D) **IHC for PIP in xenograft tumors. Percentage of cells positive for PIP was assessed using IHC and compared between each treatment group and control (CTL). * *P *<0.01 is for FLU or PD treatment versus CTL. Error Bars: ± 2SEM. AR, androgen receptor; SEM, standard error of the mean.

We next examined PIP protein expression using IHC in a cohort of twenty-four ER- breast tumors with known AR expression status [11]. ER- breast tumors were classified into AR+ and AR- subgroups as described in the Methods section and a total of twelve samples (50% of tumors) showed AR+ staining in this cohort. [11]. We then carried out IHC staining for PIP and compared the percentage of positive staining for this protein between AR+ and AR- samples. AR+ breast tumors showed a markedly higher expression of PIP (57% ± 6) compared to AR- tumors (16% ± 4), (*P *<0.01, Figures 2A and 2B). These findings suggest that AR+ staining is associated with the overexpression of PIP protein in ER- breast tumors.

### PIP is regulated *in vivo *by AR-ERK signaling

To further investigate the regulation of PIP by the AR-ERK feedback loop, we used an *in vivo *model of molecular apocrine breast cancer. Xenograft tumors were generated using MDA-MB-453 cells as described in methods. A total of four mice were studied in each of the following groups for 28 days: 1) control, 2) AR inhibition with flutamide, and 3) MEK inhibition with PD0325901. We next carried out IHC staining for PIP in the harvested tumors. Subsequently, we determined the percentage of PIP stained cells and compared the results between each treatment group and control. We observed that PIP protein expression was markedly less following flutamide and PD0325901 treatments with 3.5% ± 1 and 4.5% ± 1 of cells expressing PIP, respectively, compared to that of the control group with PIP expression in 22% ± 0.06 of cells (*P *< 0.01, Figures 2C and 2D). These findings suggest that the *in vivo *inhibition of AR and MEK result in a reduction of PIP expression in molecular apocrine tumors.

### *PIP *is a transcriptional target of CREB1

Since our data suggested that AR and ERK activation are necessary for *PIP *expression, we next investigated the regulation of *PIP *transcription by AR-ERK signaling. In this respect, we first examined the activation of *PIP *promoter by transcription factors AR and CREB1 using luciferase reporter assays. CREB1 is a well-characterized down-stream mediator of ERK signaling that we have previously shown to be a key transcription factor in regulating molecular apocrine genes *AR *and *FOXA1 *[11,20,29,30].

**Figure 3:**
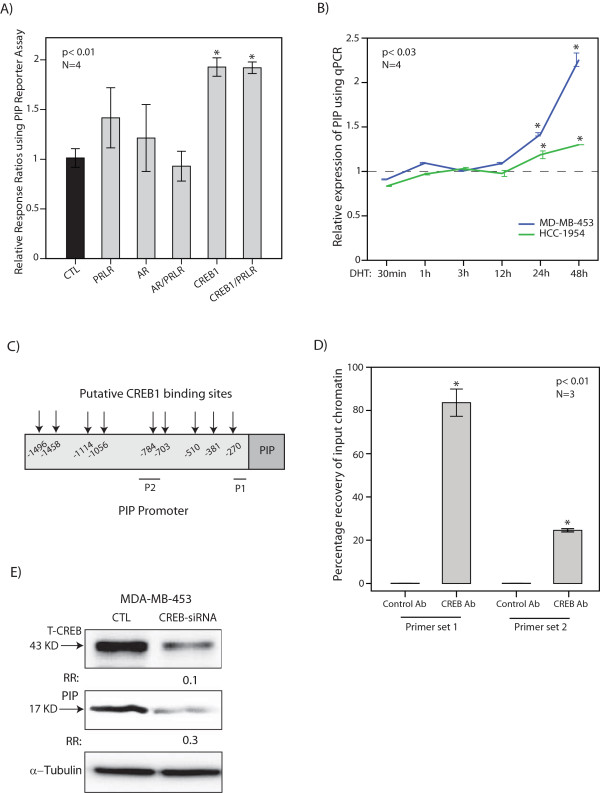
**Transcriptional regulation of PIP by AR and CREB1**. **(A) **Luciferase reporter assay. The transcriptional activation of PIP promoter by PRLR, AR, CREB1, PRLR + AR, and PRLR + CREB1 expression constructs was assessed using Dual-Luciferase assays in MCF-7 cells and relative response ratios are reported. Co-transfection with the PIP reporter vector and an empty pcDNA vector was used as a control (CTL). *P <0.01, is compared to the control group. **(B) **Induction of PIP expression following DHT treatment. PIP expression was assessed using qPCR following DHT treatment at 30 minute, 1 hour, 3 hour, 12 hour, 24 hour, and 48 hour time-points in MDA-MB-453 and HCC-1954 cell lines. Fold changes are measured relative to the respective control at each time point. *P <0.03, is compared to the control group (dashed line). Error Bars: ± 2SEM. **(C) **Putative transcription factor binding sites for CREB1 in 1.5 kb promoter region of PIP. P1 (primer set 1) and P2 (primer set 2) are regions of amplification for ChIP assays. **(D) **ChIP assay with CREB1 antibody. The results of qPCR amplification for ChIP assays are demonstrated with two sets of primers for PIP promoter. Percentage recovery of input chromatin is shown for each primer set. *, *P *<0.01 is for CREB1 Ab. versus control Ab. Error Bars: ± 2SEM. **(E) **Western blot analysis to show CREB1 and PIP protein levels following CREB1-knockdown using siRNA in MDA-MB-453 cell line. Fold changes (RR) in band densities were measured relative to non-targeting siRNA control (CTL). Ab, antibody; AR, androgen receptor; ChIP, chromatin immunoprecipitation; DHT, dihydrotestosterone; qPCR, quantitative PCR; RR, relative risk; SEM, standard error of the mean.

Due to a high degree of transfectability MCF-7 cells were used for the reporter assay experiments as described before [11,20,24]. MCF-7 cells were co-transfected with the PIP reporter vector and each of the PRLR, AR, and CREB1 expression constructs. Co-transfection with the PIP reporter vector and an empty pcDNA vector was used as a control. In addition, to test the effect of PRLR, we co-transfected this vector with each of the AR and CREB1 constructs. Forty-eight hours after the transfections reporter activities were measured and relative response ratios were calculated as described in the Methods section. We observed a significant increase in PIP reporter activity with CREB1 by approximately two-fold (*P *<0.01, Figure 3A). In addition, co-transfection of PRLR and CREB1 had a similar effect to that of CREB1 alone (Figure 3A). It is notable that AR vector, with or without PRLR co-transfection, did not significantly activate PIP promoter (Figure 3A). These results suggest that CREB1 activates PIP promoter. However, AR does not regulate the proximal 1.5 kb region of PIP promoter.

We next examined the effect of AR activation by DHT on PIP expression in MDA-MB-453 and HCC-1954 cell lines using qPCR. DHT treatments at 100 nM were carried out at 30 minute, 1 hour, 3 hour, 12 hour, 24 hour, and 48 hour time-points. For each time-point, a control experiment was carried out with cells only treated with the vehicle. Subsequently, fold change in PIP expression was calculated relative to the respective control at each time-point. We observed that PIP expression did not increase at the first 24 hour time-point following DHT treatments (Figure 3B). However, PIP expression incrementally increased at the 24 hour and 48 hour time-points, particularly in the MDA-MB-453 cell line (*P *<0.03, Figure 3B). These findings indicate that DHT treatment has a delayed effect on the induction of PIP expression in molecular apocrine cells.

Examination of the 1.5 kb PIP promoter region identified several putative binding sites for CREB1 (Figure 3C). In view of this and to assess the binding of CREB1 to the PIP promoter we carried out ChIP assays in the MDA-MB-453 cell line. Two sets of primers for the PIP promoter in proximity to the predicted binding sites were used for qPCR amplification as described in the Methods section (Figure 3C). The percentage recovery of input chromatin was calculated for each experimental set (Figure 3D). Importantly, we observed a significant enrichment for the PIP promoter region with CREB1 antibody using both primer sets (*P *<0.01, Figure 3D). Finally, we measured PIP protein expression following CREB1-knockdown in MDA-MB-453 cells. We observed that the CREB1 protein level was reduced by 90% following siRNA transfection and this resulted in an approximately 70% reduction of PIP protein expression (Figure 3E). All together, these data suggest that *PIP *is a target gene of CREB1 and the activation of AR has a delayed effect in the induction of PIP expression in molecular apocrine cells.

### PIP is necessary for cell invasion and viability

PIP is an aspartic-type protease with a specific fibronectin-degrading ability [31]. Although it is known that PIP is expressed in primary and metastatic breast cancers, the function of this protein in molecular pathogenesis of breast carcinoma remains largely unknown [32]. In order to investigate the biological significance of PIP in molecular apocrine cancer, we studied the functional effects of PIP on cell invasion and viability using the MDA-MB-453 cell line. The MDA-MB-453 line was used for the functional experiments since it represents a widely accepted cell line model for molecular apocrine subtype [5,10-12,33].

**Figure 4:**
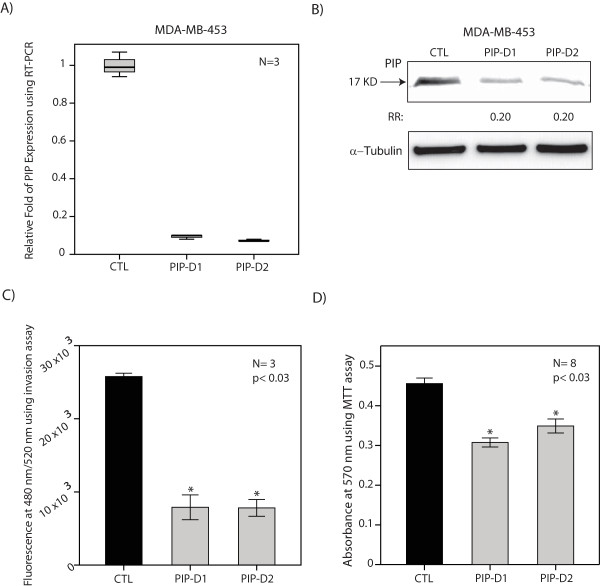
**The effect of PIP knockdown on cell invasion and viability**. **(A) **qPCR to demonstrate PIP-knockdown efficiencies with siRNA-duplex1 (D1) and siRNA-duplex2 (D2) in MDA-MB-453 cell line. PIP expression following knockdown was assessed relative to non-targeting siRNA control (CTL) and fold change is shown for each duplex. **(B) **Western blot analysis to show PIP protein level following PIP-knockdown in MDA-MB-453 cell line as described in (A). Fold changes (RR) in band densities were measured relative to the control (CTL). **(C) **The effect of PIP expression on cell invasion. Cell invasion assays were carried out after PIP-knockdown with PIP-D1 and PIP-D2 in MDA-MB-453 cell line. Transfection with non-targeting siRNA control (CTL) was used as a control. *, *P *<0.03 is for each PIP-knockdown versus CTL. Error Bars: ± 2SEM. **(D) **MTT assay to measure cell viability following PIP-knockdown with PIP-D1 and PIP-D2 in MDA-MB-453 cell line. CTL: non-targeting siRNA control. *, *P *<0.03 is for each PIP-knockdown versus CTL. Error Bars: ± 2SEM. MTT, 3-(4,5-dimethythiazol-2-yl)-2,5-diphenyl tetrazolium bromide; qPCR, quantitative PCR; RR, relative risk; SEM, standard error of the mean.

To test the functional effects of PIP we carried out PIP-knockdown in MDA-MB-453 cells using two siRNA duplexes as described in the Methods section. The efficiency of knockdowns was assessed by qPCR and western blot analysis. Importantly, we observed an approximately 90% reduction in PIP transcription and 80% reduction in PIP protein level following PIP-knockdown with both siRNA duplexes (Figures 4A and 4B).

We first examined whether PIP expression is required for cell invasion in molecular apocrine cells. Cell invasion was assessed using a basement membrane, fluorometric cell invasion assay kit as described in the Methods section. Invasion assays were carried out in three biological replicates for each of the following groups: 1) control-siRNA, 2) PIP-siRNA duplex1 (PIP-D1), and 3) PIP-siRNA duplex2 (PIP-D2). Subsequently, fluorescence measurements at 480 mm/520 mm were compared between PIP-knockdown and control groups. Notably, there was a marked reduction in cell invasion by approximately three-fold following PIP-knockdown with both duplexes compared to the control group (*P *<0.03, Figure 4C). We next assessed the effect of PIP expression on cell viability. MDA-MB-453 cells were studied in PIP-D1, PIP-D2, and control-siRNA groups and cell viability was assessed using MTT assay seventy-two hours after siRNA transfections. We observed a 30% to 40% reduction in cell viability following PIP-knockdown compared to the control group (*P *<0.03, Figure 4D). These findings suggest that PIP expression is necessary for cell invasion and viability in molecular apocrine cells.

#### PIP is necessary for the activation of ERK and Akt signaling

To investigate an underlying mechanism for the effect of PIP on cell viability, we examined the signaling consequences of PIP-knockdown in molecular apocrine cells. PIP-knockdown was carried out using PIP-D1 and PIP-D2 in the MDA-MB-453 cell line and non-targeting siRNA was used as a control. Seventy-two hours after transfections protein lysates were extracted for western blot analysis. We first studied the effect of PIP-knockdown on the phosphorylation of ERK and Akt, since these phosphorylations are key signaling events in cell proliferation [34].

**Figure 5:**
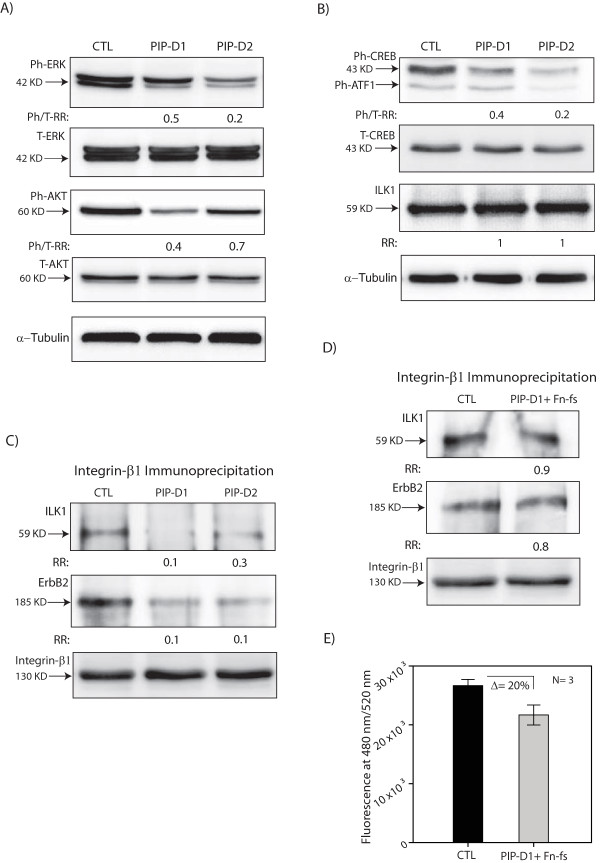
**The effect of PIP knockdown on ERK-Akt and integrin-β1signaling**. **(A) **Western blot analysis to measure the levels of phosphorylated (Ph)-ERK, total (T)-ERK, ph-Akt, and T-Akt following PIP-knockdown with siRNA duplex1 (PIP-D1) and duplex2 (PIP-D2) in MDA-MB-453 cell line. Fold changes of Phospho/Total ratios (Ph/T-RR) were assessed relative to non-targeting siRNA control (CTL). **(B) **Western blot analysis to measure the level of ph-CREB1, T-CREB1, and ILK1 following PIP-knockdown as described in (A). Ph-ATF1 is the phosphorylated form of CREB-related protein that is known to be detected by this antibody. **(C) **Integrin-β1 immunoprecipitation (IP). IP assays were carried out with Integrin-β1 following PIP-knockdown with PIP-D1 and PIP-D2 in MDA-MB-453 cell line. Non-targeting siRNA was used as control (CTL). Western blot analysis was carried out on IP samples to measure the integrin-β1 binding to ILK1 and ErbB2. Immunoblotting with integrin-β1 antibody was used as a loading control. Fold changes (RR) of ILK1 and ErbB2 following PIP-knockdown were measured relative to that of control-siRNA. **(D) **Integrin-β1 immunoprecipitation following PIP-knockdown and the addition of fibronectin fragments (Fn-fs). PIP-knockdown with PIP-D1 was carried out as described in (C). Twenty-four hours after PIP-knockdown, cells were treated with α-chymotryptic fibronectin fragment 120K at 100 µg/ml concentration. Control cells were treated with vehicle only. Fold changes (RR) of ILK1 and ErbB2 following PIP-knockdown + Fn-fs were measured relative to the control. **(E) **The effect of fibronectin fragments on cell invasion following PIP-knockdown. Cell invasion assays were carried out after PIP-knockdown with PIP-D1 in MDA-MB-453 cell line. Transfection with non-targeting siRNA control (CTL) was used as a control. Treatment with fibronectin fragments was carried out as described in (D). Error Bars: ± 2SEM. Δ; is the difference between CTL and PIP-D1+Fn-fs groups. ERK, extracellular signal-regulated kinase; ILK1, integrin-linked kinase 1; RR, relative risk; SEM, standard error of the mean.

Following western blot analysis, fold changes in phospho-ERK/total-ERK and phospho-Akt/total-Akt ratios were measured in PIP-knockdown relative to the control. Notably, there was a marked reduction in phospho-ERK/total-ERK ratio between 0.2- and 0.5-fold following PIP-knockdown (Figure 5A). Similarly, PIP-knockdown resulted in a 0.4- to 0.7-fold reduction of phospho-Akt/total-Akt ratio (Figure 5A). We next assessed the effect of PIP-knockdown on the phosphorylation of CREB1. CREB1 is a critical downstream mediator of the EGFR-ErbB2 pathway, which is activated by both Akt and ERK signaling [29,35,36]. Fold change in phospho-CREB1/total-CREB1 ratio was measured in PIP-knockdown relative to the control. Consistent with phospho-ERK and phospho-Akt data, we observed a marked reduction in phospho-CREB1/total-CREB1 ratio between 0.2- and 0.4-fold following PIP-knockdown (Figure 5B). These findings suggest that PIP expression is necessary to maintain the phosphorylation of ERK, Akt, and their downstream target CREB1 in molecular apocrine cells.

### PIP is necessary for integrin-β1 binding to ILK1 and ErbB2

Enzymatic degradation of fibronectin releases fragments that bind to integrin-β1 and activate intracellular signaling by its cytoplasmic tail [37,38]. It is known that the activation of integrin-β1 promotes cell adhesion and invasion [37,39]. In addition, integrin-β1 activation induces some of the key signaling pathways such as MAPK/ERK and PI3K/Akt that are involved in cell proliferation [37,40]. Since it is known that PIP is a protease with fibronectin-degrading ability [31], we hypothesized that PIP may be required for the integrin-β1 activation in molecular apocrine cells.

Integrin-β1 activation by fibronectin fragments leads to the binding of this receptor to its binding partners. One of the integrin-β1 key binding partners is integrin-linked kinase 1 (ILK1), which binds to the activated integrin-β1 and mediates downstream signaling effects such as the activation of Akt [41,42]. Therefore, we investigated the effect of PIP-knockdown on the binding between integrin-β1 and ILK1 using an IP assay. Transfections of PIP-D1 and PIP-D2 were carried out in the MDA-MB-453 cell line and non-targeting siRNA was used as a control. Seventy-two hours after siRNA transfections, cells were lysed for IP and immunoblotting assays. It is notable that ILK1 protein levels were similar in the extracted lysates among PIP-knockdown and control experiments (Figure 5B). We next carried out an IP assay with integrin-β1 antibody and subjected the samples to western blot analysis using ILK1 antibody. Immunoblotting of IP samples using integrin-β1 antibody was used as a loading control. Importantly, there was a 70% to 90% reduction in binding of integrin-β1 to ILK1 following PIP-knockdown compared to the control (Figure 5C).

Moreover, it has previously been reported that integrin-β1 binds to ErbB2 in human carcinoma cell lines [43]. Since ErbB2 overexpression is present in the majority of molecular apocrine tumors, we examined the association between integrin-β1 and ErbB2 in MDA-MB-453 cells and evaluated a possible role for PIP expression in this process. Following IP assays in PIP-knockdown and control-siRNA samples, we carried out western blot analysis using ErbB2 antibody. Notably, we observed that integrin-β1 binds to ErbB2 in the control experiment and this binding was decreased by 90% following PIP-knockdown compared to the control (Figure 5C).

Finally, we asked whether the effects of PIP-knockdown in the reduction of integrin-β1 binding to ILK1 and ErbB2 can be reversed by fibronectin fragments. This was assessed by the addition of α-chymotryptic fibronectin fragment 120K at 100 µg/ml concentration 24 hours after transfection of MDA-MB-453 cells with PIP-D1. Transfection with non-targeting siRNA and treatment with vehicle only was used as a control. Following the extraction of lysates and IP assays we subjected the samples to western blotting using ILK-1 and ErbB2 antibodies. Immunoblotting with integrin-β1 antibody was applied as a loading control. Importantly, we observed a nearly complete recovery of integrin-β1 binding to ILK1 and ErbB2 in PIP-knockdown experiments following the addition of fibronectin fragments to levels similar to that of the control (Figure 5D). Moreover, the addition of fibronectin fragments reversed the reduction of cell invasion observed following PIP-knockdown to approximately 80% of the control levels (Figure 5E). All together, these data suggest that PIP expression is necessary for integrin-β1 binding to ILK1 and ErbB2 in a process that is mediated through the fragmentation of fibronectin.

## Discussion

Molecular apocrine is one of the major subtypes of ER- breast cancer that is characterized by the overexpression of a steroid-response gene signature [3-5]. Investigation of key functional pathways in this subtype of breast cancer is an essential step for the discovery of effective therapeutic strategies in this disease. We have recently identified a positive feedback loop between the AR and ERK signaling pathways in the molecular apocrine subtype that is mediated through ErbB2 and CREB1 [11]. In this study, we demonstrated that this AR-ERK feedback loop regulates the transcription of some of the top ranking genes in the molecular apocrine signature (Figure 1A and Table 2). Among these genes, we observed that *PIP*, *DUSP6*, *S100A8*, and *FOXA1 *expression were consistently reduced following the inhibition of AR-ERK signaling.

DUSP6 is a specific ERK phosphatase that is highly regulated by ERK at the promoter level mediated through ETS1, a well known target of activated ERK [44]. This phenomenon explains the significant reduction of DUSP6 expression following the inhibition of AR-ERK signaling. In addition, the presence of *DUSP6*, a gene closely regulated by ERK signaling, among the top ranking genes in the molecular apocrine signature provides further support for the importance of ERK activation in this subtype of breast cancer. *FOXA1 *is another highly regulated gene by AR-ERK signaling that has been the subject of intense interest because of its emerging role as a critical modulator of ER and AR function [33,45,46]. In addition, we have recently identified a cross-regulation network between FOXA1 and ErbB2 signaling that connects FOXA1 to some of the key signaling pathways in ER- breast cancer [20]. Moreover, we observed that S100A8 expression is regulated by the modulation of AR-ERK. S100A8 and its isoform S100A9 form a secreted protein complex that is involved in inflammation, cell invasion and migration [47]. The observed regulation of S100A8 by AR-ERK signaling is in agreement with a previous study that demonstrated a positive feedback loop between Ras-activated ERK and S100A8 expression [48]. Importantly, in our study *PIP *was the most regulated molecular apocrine gene by AR-ERK signaling and, therefore, we investigated the biological significance of this gene in the molecular apocrine subtype.

PIP is a secreted protein with aspartic-type protease activity specific to fibronectin [31]. Several studies have shown that PIP protein is overexpressed in primary and metastatic breast cancers with a possible prognostic value in this disease [32,49-51]. Despite these findings, the functional role of PIP in breast cancer has remained largely unknown. Our findings suggest that PIP is overexpressed in ER-/AR+ breast tumors and PIP expression is highly regulated by AR-ERK signaling in both *in vitro *and *in vivo *molecular apocrine models (Figures 1 and 2). Considering that the majority of molecular apocrine tumors have luminal features [52], the PIP expression pattern in ER- breast tumors may contribute to the biological differences observed between the luminal and basal subtypes of ER- breast cancer. It is notable that PIP protein expression has been associated with apocrine histological differentiation [49], and, therefore, the overexpression of PIP represents a common feature between molecular apocrine subtype and apocrine histological classification.

The regulation of PIP expression by the AR-ERK feedback loop is explained by the fact that *PIP *is a CREB1 target gene and is induced by AR activation (Figure 3). CREB1 is a well-characterized ERK signaling transcription factor that is a down-stream target of active ERK through the mediation of the RSK and MSK family of kinases [29,30,35,53,54]. Importantly, *AR *itself is a CREB1 target gene that activates the ERK-CREB1 axis through the induction of ErbB2 expression [11]. Therefore, the transcriptional regulation of PIP is mediated by a positive feedback loop between AR and CREB1 in molecular apocrine cells. Furthermore, we have recently demonstrated that the molecular apocrine gene *FOXA1 *is also a CREB1 target gene [11,20]. All together, these findings suggest that the ERK-CREB1 axis has a key role in the transcriptional regulation of the molecular apocrine genes.

Moreover, transcriptional regulation of PIP by AR has been previously studied [55-57]. In general, hormonal regulation of PIP expression is a complex process that involves gene structure differences and tissue-specific transacting factors [32]. For example in ER+ cell lines, STAT5 and Runx2 cooperate with AR to stimulate PIP transcription [56,57]. Our results suggest that AR does not directly activate the proximal 1.5 kb region of the PIP promoter. However, it is possible that AR activation of PIP can be mediated through a more distant site. In fact, it has recently been shown that Runx2 and AR co-regulate an enhancer site 11 kb upstream of the PIP transcription start site [57]. Moreover, we observed a delayed pattern of PIP induction in molecular apocrine cells following the activation of AR by DHT starting at the 24 hour time-point (Figure 3B). This represents a different pattern of induction compared to that observed with some of the other AR-activated genes, such as PSA and ErbB2, that demonstrate a rapid increase in expression within 12 hours of DHT treatment [11,58]. It is notable that a delayed induction of PIP following DHT has been previously reported in other studies [59], and may indicate the time necessary for the activation of other signaling pathways needed in the stimulation of PIP transcription.

**Figure 6:**
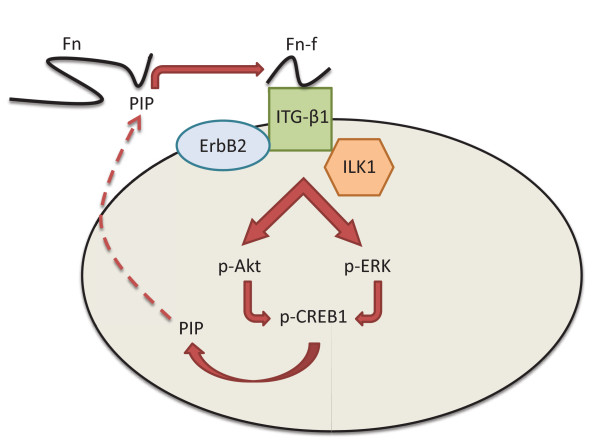
**Schematic diagram of the PIP signaling pathway in ER-negative breast cancer**. Red arrow denotes stimulatory effect. ER, estrogen receptor; Fn, fibronectin; Fn-f, Fibronectin fragment; ITG-β1, integrin-β1.

Our study suggests that there is a feedback loop between PIP and ERK-Akt signaling in molecular apocrine cells (Figure 6). Following the induction of PIP expression by CREB1, the secreted PIP mediates protease degradation of fibronectin to fragments, which results in the activation of integrin-β1 signaling (Figures 5 and 6). Importantly, in the absence of PIP there is a marked reduction of integrin-β1 binding to its binding partners ILK1 and ErbB2 that can be reversed by the addition of fibronectin fragments (Figures 5C and 5D). ILK1 is a key binding partner of the activated integrin-β1 receptor that mediates the induction of Akt and ERK signaling pathways [41,42,60]. In addition, integrin-β1 is associated with the EGFR-ErbB2 receptor family and mediates an EGF-independent activation of the EGFR-ErbB2 signaling pathway, which in turn results in the induction of MAPK/ERK signaling and cell proliferation [27,43,61]. In fact, we observed a marked reduction in the phosphorylation levels of ERK, Akt, and their downstream target CREB1 following PIP-knockdown in molecular apocrine cells (Figures 5A and 5B). Since *PIP *is a CREB1 target gene, this regulation of CREB1 phosphorylation by PIP provides a positive feedback loop mechanism between PIP and CREB1 mediated through the integrin-ERK and integrin-Akt signaling pathways (Figure 6).

The functional significance of PIP is evident by the fact that PIP expression is necessary for cell invasion and viability in molecular apocrine cells (Figure 4). Notably, a three-fold reduction in cell invasion observed following PIP-knockdown indicates that the secretion of this protein has a key role in maintaining the invasive properties of molecular apocrine cells. These functional effects can be explained by the positive regulatory role of PIP on the integrin-ERK and integrin-Akt signaling pathways mediated through the generation of fibronectin fragments (Figure 6). Interestingly, secretion of other fibronectin-degrading enzymes such as neutral serine proteases have been reported in T-cell lymphomas [62], suggesting that a similar process may be involved in the invasion of other malignant cells. Furthermore, our findings regarding the effect of PIP expression on cell viability is consistent with a recent study that demonstrated a decrease in the proliferation of the ER+ cell line T47D following PIP down-regulation [57].

## Conclusions

In summary, we have characterized the PIP signaling pathway in molecular apocrine breast cancer (Figure 6). We demonstrated that PIP expression is necessary for the activation of integrin-β1 signaling and the induction of the ERK and Akt signaling pathways as well as their downstream target CREB1. Furthermore, we showed that *PIP *is a CREB1 target gene and, therefore, there is positive feedback loop between PIP and CREB1 signaling.

Importantly, PIP expression has a profound effect in maintaining cell invasion and viability of molecular apocrine cells. These findings offer the tantalizing possibility of exploiting PIP signaling as a potential therapeutic target in molecular apocrine breast cancer.

## Abbreviations

AR: androgen receptor; ChIP: chromatin immunoprecipitation; DHT: dihydrotestosterone; (D)MEM: (Dulbecco's) modified Eagle's medium; EGF: epidermal growth factor; ER: estrogen receptor; ERK: extracellular-signal-regulated kinase; FBS: fetal bovine serum; IHC: immunohistochemistry; ILK1: integrin-linked kinase 1; IP: immunoprecipitation; MEK: mitogen***-***activated protein kinase kinase*; *MAPK: mitogen-activated protein kinase; MTT: 3-(4,5-dimethythiazol-2-yl)-2,5-diphenyl tetrazolium bromide; PRL: prolactin; PRLR: prolactin receptor clone; qPCR: quantitative polymerase chain reaction; SEM: standard error of the mean; siRNA: small interfering RNA.

## Competing interests

The authors declare that they have no competing interests.

## Authors' contributions

AN conceived the study, designed the experiments, and drafted the manuscript. AN and MM carried out the experiments. All authors read and approved the final manuscript.
